# Permanent His‐Bundle pacing in a patient with advanced conduction system disease: What is the mechanism of QRS narrowing?

**DOI:** 10.1002/ccr3.1569

**Published:** 2018-05-15

**Authors:** Nishant Verma, Bradley P. Knight

**Affiliations:** ^1^ Division of Cardiology Department of Medicine Feinberg School of Medicine Northwestern University Chicago IL USA

**Keywords:** cardiac resynchronization, longitudinal dissociation, pacemaker, permanent His‐bundle pacing

## Abstract

QRS narrowing during permanent His‐bundle pacing is primarily thought to result from longitudinal dissociation within the His bundle. We present a case with an alternative mechanism, highlighting the likelihood that there are actually multiple explanations for this phenomenon. In addition, this case highlights the utility of His‐bundle pacing even in the face of a wide QRS.

## 1 CASE REPORT

**Figure 1:**
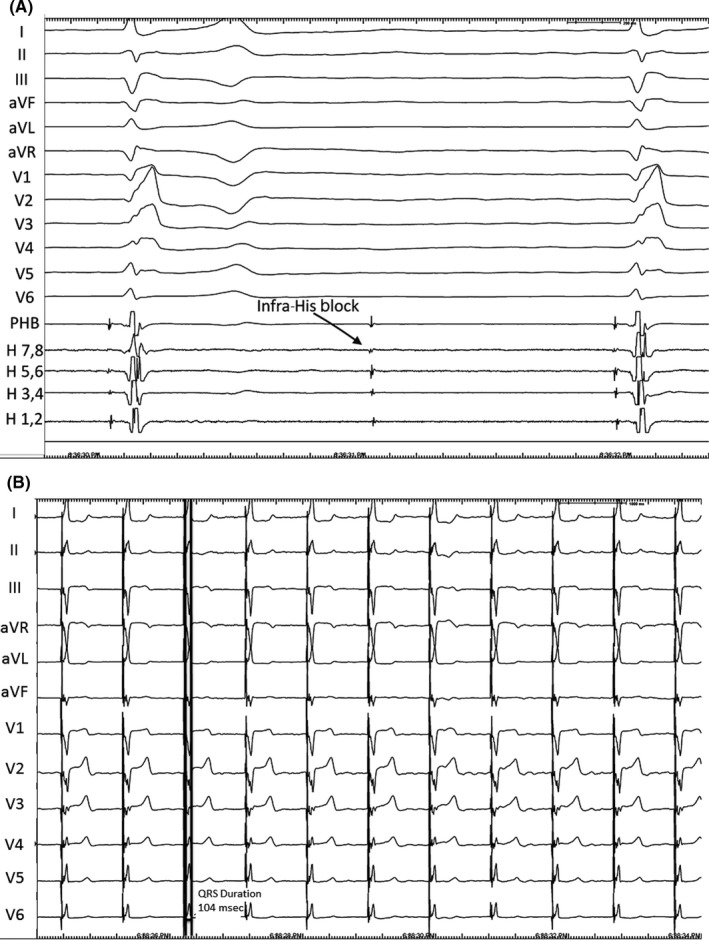
A, Baseline 12‐lead electrocardiogram (ECG) and intracardiac electrograms. The patient's baseline 12‐lead ECG on admission for syncope showed atrial fibrillation with a slow ventricular response. The baseline QRS duration was 156 ms and a right bundle branch block (RBBB) with left anterior fascicular block (LAFB). The baseline intracardiac electrograms during electrophysiology study and permanent his bundle (PHB) lead implantation are shown. The octapolar His (H) catheter placed via the right femoral vein is displayed. These show that infra‐Hisian block results in the slow ventricular rate. Pacing with the octapolar catheter resulted in QRS narrowing so a PHB lead was implanted. The electrogram from the PHB lead is displayed on the EP laboratory recording system as shown. B, 12‐Lead ECG during high‐output unipolar pacing from permanent His‐bundle lead. High‐output unipolar pacing from the PHB lead results in resolution of the conduction disease with QRS normalization with a duration of 104 ms. On the intracardiac electrograms recorded at this time, the His potentials that were recorded on both the octapolar His catheter and PHB lead were not captured although the QRS normalized

An 85‐year‐old man with coronary artery disease (CAD), prior coronary artery bypass graft (CABG) surgery and permanent atrial fibrillation (AF) was admitted with recurrent, unexplained syncope. His baseline electrocardiogram (ECG) and telemetry monitoring showed AF with a slow ventricular rate as well as right bundle branch block (RBBB) and left anterior fascicular block (LAFB; Figure 1A). The QRS duration was 156 milliseconds. Due to concern over paroxysmal AV block or ventricular arrhythmia as the cause of his syncope, electrophysiology (EP) study with possible device implantation was recommended. An octapolar catheter (Biosense Webster, Diamond Bar, CA) was positioned near the His‐bundle location. Intracardiac electrograms (EGMs) revealed infra‐Hisian block at baseline, and there were no inducible ventricular arrhythmias during the EP study (Figure 1A). Pacing from the His catheter in this location resulted in QRS narrowing, so the decision was made to implant a permanent His‐bundle (PHB) pacemaker. The octapolar catheter was left in the His‐bundle position as a fluoroscopic marker for PHB lead placement.

**Figure 2:**
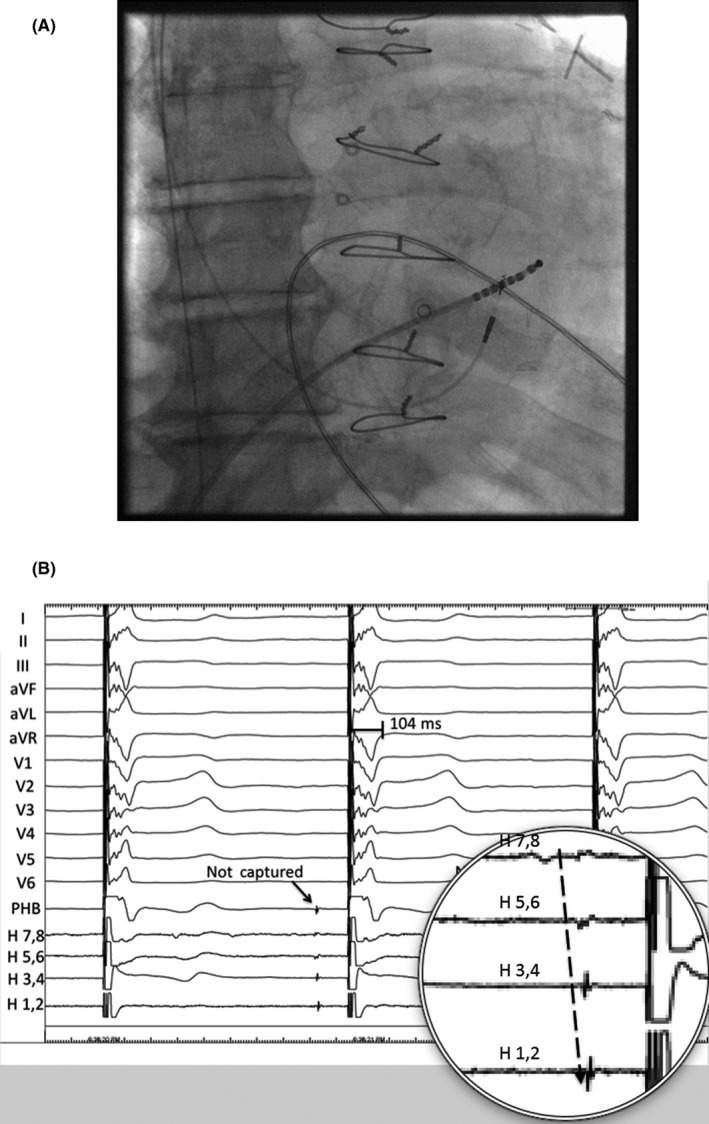
A, Octapolar catheter position relative to permanent His‐bundle lead. Fluoroscopy of the relative positions of the octapolar His catheter and permanent His‐bundle lead is shown after repositioning of the octapolar catheter to record a His or proximal right bundle signal distant to the PHB lead. B, Intracardiac electrograms during permanent His‐bundle lead pacing with a narrow QRS. These intracardiac electrograms were recorded during PHB lead pacing at an output that resulted in QRS normalization. The His signal recorded on the PHB lead and the signal distal to the lead recorded on the octapolar His (H) catheter are both not captured despite QRS narrowing (solid arrow). A closer look at the octapolar His catheter recordings shows antegrade conduction (dotted arrow). These tracings indicate that recruitment of the recorded His bundle and more distal right bundle electrograms are not necessary for QRS normalization in this case, a finding that is not consistent with longitudinal dissociation of the His bundle

A fixed‐curve delivery sheath (His C315, Medtronic, Inc.) was used to direct the PHB lead (Model 3830, Medtronic, Inc.) toward the membranous septum. The PHB lead was connected to the EP laboratory recording system (Cardiolab, GE), and a His potential was recorded from the PHB lead (Figure 1A). High‐output unipolar pacing from the PHB lead at this location resulted in a narrow QRS complex (104 milliseconds) with resolution of the RBBB and LAFB (Figure 1B). However, despite resolution of the conduction abnormalities, intracardiac EGMs on the octapolar catheter revealed that the recorded His‐bundle potential was not captured during PHB lead pacing. The octapolar catheter was advanced beyond the PHB lead tip to ensure there was not capture of the distal His bundle (or right bundle branch; Figure 2A). Recordings from the octapolar catheter in this location revealed antegrade activation of the His bundle, again without capture of this electrogram during pacing (Figure 2B). Pacing at lower outputs resulted in non‐selective His capture with a His threshold of 1.75 V @ 1.0 milliseconds and an RV septal threshold of 0.75 V @ 1.0 milliseconds. The thresholds remained stable, and the patient was discharged the next morning. What is the mechanism of QRS narrowing in this patient with advanced conduction disease?

## 2 DISCUSSION

Previous discussions regarding QRS narrowing with permanent His‐bundle pacing have focused on the concept of longitudinal dissociation of the His bundle. This was first described by Narula.^1^ Extensive histologic studies had shown that there was discrete collagen separation of Purkinje strands within the His bundle, possibly indicating that individual fibers were predestined to their locations in the bundle branches.^2^ Therefore, intra‐Hisian disease, rather than distal conduction disease, could result in a QRS with a bundle branch block morphology. This was thought to explain the frequent coexistence of right bundle branch block and left anterior fascicular block as the 2 structures travel close together within the His bundle.^3^ In patients with left bundle branch block (LBBB) or isolated left axis deviation (LAD), pacing at a proximal His‐bundle site resulted in reproduction of the baseline LBBB while pacing distal to that site resulted in a narrow QRS complex. This study formed the conceptual understanding of what had previously been a solely anatomic theory. More recent reports have validated the concept of QRS narrowing in the presence of significant conduction disease during PHB pacing either by pacing distal to the site of block or recruiting distal tissue with increased output.^4^, ^5^ Other theories to explain this phenomenon have used models of cardiac rhythm based on cable electronics theory. These include virtual electrode polarization and differential source‐sink relationships.^6^, ^7^ It remains unclear which of these is the true mechanism of QRS narrowing although the most widely accepted one is the concept of longitudinal dissociation.

The present case, however, demonstrates QRS narrowing and a reversal of RBBB despite a lack of recruitment of the recorded His‐bundle (or proximal right bundle) potential. A possible explanation for this finding could be that the portion of the right bundle that is captured is further distal than what is recorded. In this case, this seems exceedingly unlikely given the large distance between the distal end of the octapolar catheter and the PHB lead after the catheter was moved further distally to record the right bundle.

Theoretically, capture of the left‐sided His could somehow result in QRS narrowing without involvement of the right‐sided His bundle. This would require distal connections between the left and right bundles; however, these types of connections have not been described in humans, making this an unlikely explanation. In this case, the recorded His potential was activated in antegrade fashion rather than retrograde fashion, although site of turnaround could be more distal than to what is recorded. Left‐sided His recordings during PHB pacing would be a useful method of ruling this possibility in or out.

**Figure 3:**
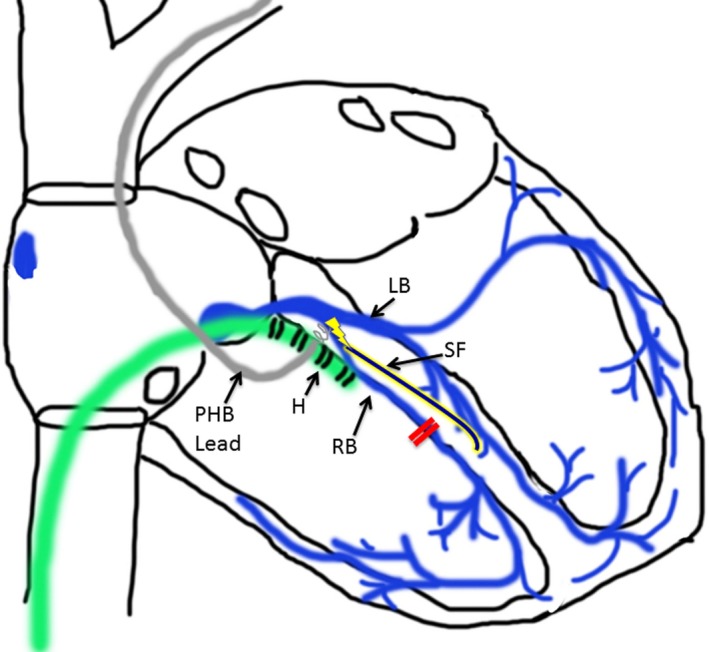
Mechanism for QRS narrowing via a septal fascicle. This figure shows the purported mechanism of QRS narrowing in this case. Early branching, an anatomic variant of the right bundle (RB), functioning as a septal fascicle (SF) allows for conduction (lightning bolt) from the permanent His‐bundle (PHB) lead to bypass the distal his/RB potential recorded on the His (H) catheter and the blocked left anterior fascicle, insert more distally, and overcome the baseline right bundle branch block (red lines) to allow for QRS narrowing and normalization of the QRS axis

It is also possible that the His signals that are recorded on the decapolar catheter are separate from the RB potentials which are actually recruited and captured. Anatomically, the right bundle is thought to be a discrete structure; however, multiple variations exist which likely occur more commonly than is appreciated. Early branching of the right bundle, duplication of the right bundle, Lancisi fibers, and fasiculoventricular tracts are all described anatomic variants, which behave functionally like a superior septal fascicle.^8^, ^9^ Capture of any one of these structures could result in QRS narrowing without capture of the true His bundle or proximal right bundle. A rare atypical pathway such as a nodofasicular tract could also theoretically bypass the His bundle and insert into the distal conduction system but would be expected to result in a wide QRS complex. In this particular case, the presence of one of the anatomic variants of the right bundle, including early branching, behaving as an additional septal fascicle is the most likely explanation for the QRS narrowing that is observed. In such a case, the proximal end of the fascicle would exit distal to the left anterior fascicle, accounting for the change in mean QRS axis seen with high‐output unipolar pacing (Figure 3). As this case highlights, the mechanism of QRS narrowing with PHB pacing in patients with a wide QRS complex can be complex and there are multiple possible explanations in addition to the commonly proposed mechanism of longitudinal dissociation.

## AUTHORSHIP

NV: Primary author, wrote and edited the manuscript. BPK: wrote and edited the manuscript.

## CONFLICT OF INTEREST

Dr. Verma receives honoraria from Medtronic, Inc. Dr. Knight is a consultant for and receives honoraria from Medtronic, Inc.
